# Rapid screening and identification of viral pathogens in metagenomic data

**DOI:** 10.1186/s12920-021-01138-z

**Published:** 2021-12-14

**Authors:** Shiyang Song, Liangxiao Ma, Xintian Xu, Han Shi, Xuan Li, Yuanhua Liu, Pei Hao

**Affiliations:** 1 Key Laboratory of Molecular Virology and Immunology, Institut Pasteur of Shanghai, Center for Biosafety Mega-Science, Chinese Academy of Sciences, Shanghai, 200031 China; 2 Bio-Med Big Data Center, Key Laboratory of Computational Biology, CAS-MPG Partner Institute for Computational Biology, Shanghai Institutes for Biological Sciences, Shanghai Institute of Nutrition and Health, Chinese Academy of Sciences, Shanghai, 20031 China; 3 Key Laboratory of Synthetic Biology, CAS Center for Excellence in Molecular Plant Sciences, Chinese Academy of Sciences, Shanghai, 200032 China

**Keywords:** Pathogen screening, Metagenomics data, Epidemic, SARS-CoV-2, Viral genome assembly

## Abstract

**Background:**

Virus screening and viral genome reconstruction are urgent and crucial for the rapid identification of viral pathogens, i.e., tracing the source and understanding the pathogenesis when a viral outbreak occurs. Next-generation sequencing (NGS) provides an efficient and unbiased way to identify viral pathogens in host-associated and environmental samples without prior knowledge. Despite the availability of software, data analysis still requires human operations. A mature pipeline is urgently needed when thousands of viral pathogen and viral genome reconstruction samples need to be rapidly identified.

**Results:**

In this paper, we present a rapid and accurate workflow to screen metagenomics sequencing data for viral pathogens and other compositions, as well as enable a reference-based assembler to reconstruct viral genomes. Moreover, we tested our workflow on several metagenomics datasets, including a SARS-CoV-2 patient sample with NGS data, pangolins tissues with NGS data, Middle East Respiratory Syndrome (MERS)-infected cells with NGS data, etc. Our workflow demonstrated high accuracy and efficiency when identifying target viruses from large scale NGS metagenomics data. Our workflow was flexible when working with a broad range of NGS datasets from small (kb) to large (100 Gb). This took from a few minutes to a few hours to complete each task. At the same time, our workflow automatically generates reports that incorporate visualized feedback (e.g., metagenomics data quality statistics, host and viral sequence compositions, details about each of the identified viral pathogens and their coverages, and reassembled viral pathogen sequences based on their closest references).

**Conclusions:**

Overall, our system enabled the rapid screening and identification of viral pathogens from metagenomics data, providing an important piece to support viral pathogen research during a pandemic. The visualized report contains information from raw sequence quality to a reconstructed viral sequence, which allows non-professional people to screen their samples for viruses by themselves (Additional file 1).

**Supplementary Information:**

The online version contains supplementary material available at 10.1186/s12920-021-01138-z.

## Background

In the last 20 years, many disease outbreaks have been attributed to viruses, from SARS, Ebola [1, 2], MERS [3], Zika [4], to the recent pneumonia of SARS-CoV-2. With faster transportation and globalization, the impacts of an emerging viral outbreak have become more severe. We are only a year from the first reported pneumonia case caused by SARS-CoV-2, the disease ultimately caused a world-wide epidemic. As of November 1, 2021, the pandemic has led to more than 247,000,000 cases and 5,000,000 deaths. The basic reproductive number (R0) of COVID-19 ranges from 2–3.5 during the early phase, which is higher than SARS and MERS regardless of the prediction model [5]. To better understand and eventually prevent such viral outbreaks, it is critical to identify viral pathogens and obtain their genetic sequences in a timely manner. This is the basis for tracing their sources, and understanding the molecular mechanisms of pathogen infection, transmission, and evolution.

Next-generation sequencing (NGS) techniques have recently switched from proof-of-concept studies to a routinely used tool in the clinical microbiology laboratory. It is now easy and efficient to generate metagenomics data for direct analysis of genetic materials in order to identify viruses and their abundances in various environments [6]. However, with the ease of metagenomics data generation, the downstream data analysis still faces challenges, including computational identification of viral species and their complete genome sequences in a fast yet accurate manner from hundreds of millions of short reads.

Bioinformatics tools used for virus identification either use amino acid profile (gene-based) approaches or nucleotide profile (reference-based) approaches. Gene-based identification methods are fast but not suitable for viruses with unknown marker genes [7]. Reference-based methods are more informative but time-consuming for large metagenomics data when de novo assembly is involved. Furthermore, incorrect information during assembly may mislead the results [8, 9]. Fortunately, there are existing tools that can directly align short reads to the reference database, rapidly identify viruses, and make full use of the information in the whole genome [10–13]. In particular, to accelerate the speed, Tithi et al. [13] developed a tool called the FastViromeExplorer, which has been proven to be fast and accurate for identifying and quantifying viruses in metagenomics data. The FastViromeExplorer employs Kallisto, a pseudo-alignment tool that maps input reads into the reference database and makes reads mapping both lightweight and fast.

Since the SARS-CoV-2 outbreak, scientists from different fields have contributed various features to better understanding the novel coronavirus. At the initial stage, most researchers have focused on the viral genomes (i.e., tracing the origin of SARS-CoV-2 through genome sequence comparisons with a viral reference database [14, 15]; screening antiviral drugs by matching spatial structures between drugs and key viral proteins predicted and annotated from the genome [16]). For the sake of pathogen surveillance, the viral genomes are increasingly important for understanding viral pathogen characteristics. As we stated above, when de novo assembly was considered, virus detection and identification suffered from incorrectness and heavy computational costs. Therefore, insufficient coverage of individual genomes, high variation within the same species, and frequent occurrence of repetitive regions constitute the main problems. In comparison to de novo genome construction, de novo reference-guided assembly uses long-read sequencing combined with a high-performance algorithm. While more and more viral genome sequences need further investigation with regard to genome-wide mutations at both nucleotide and amino acid levels, de novo reference-guided assembly assesses the impact of genetic variations and rearrangements on evolution, studying genetic responses to environmental changes [17]. Further, it aids in the performance of the genome-wide linkage disequilibrium analysis, which is based on study population histories and identifies signatures of selection in natural populations or admixture event timing [18].

**Fig. 1:**
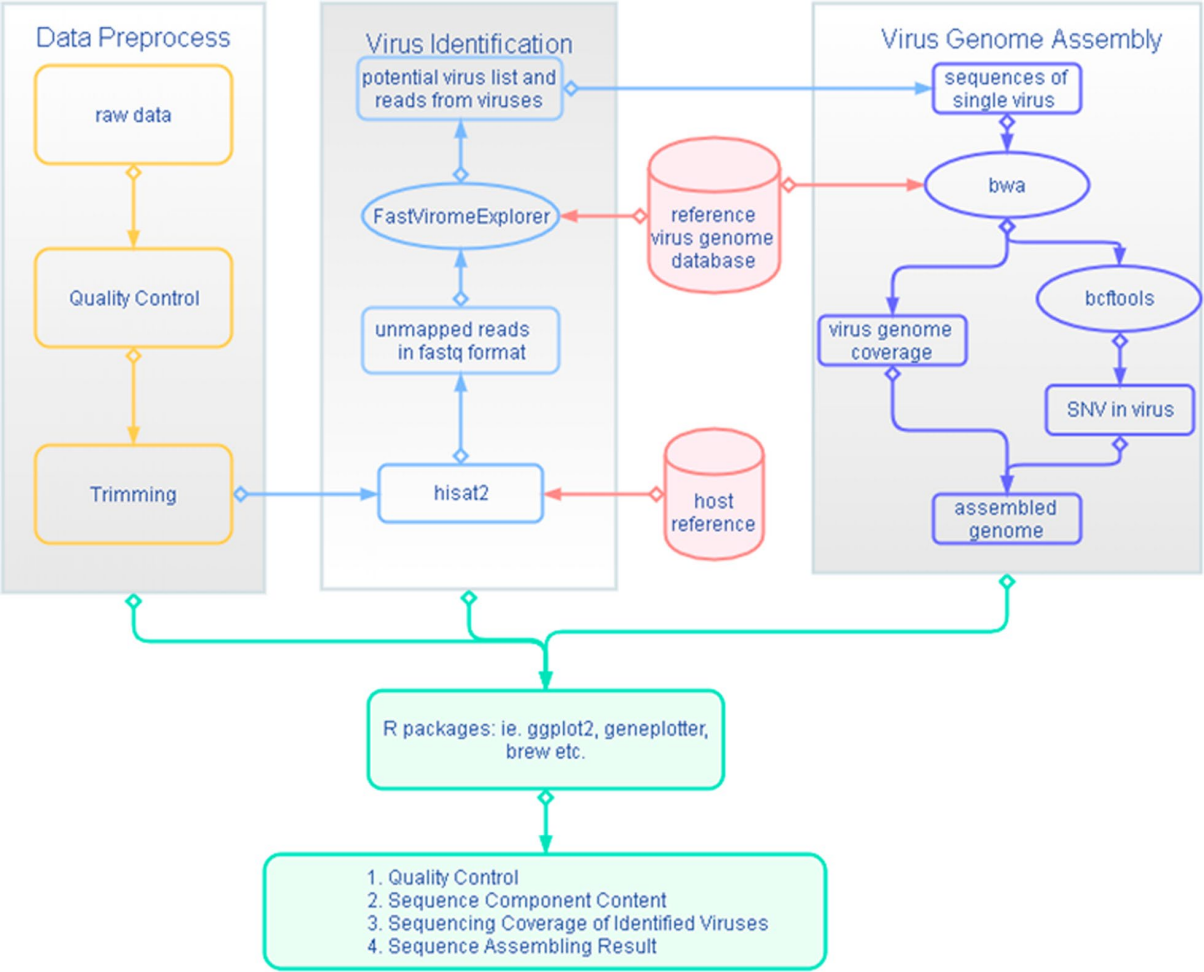
Viral pathogens Identification Workflow. VIW is consisted of four modules, (1) data preprocessing; (2) virus detection; (3) viral genome assembly and (4) report generation

In this paper, we present a fast and accurate workflow, virus identification workflow—VIW, for virus identification and genome assembly based on NGS data to support viral pathogen research, especially during the COVID-19 pandemic. This workflow consists of four modules (Fig. 1): (1) data preprocessing of NGS data; (2) virus detection using the FastViromeExplorer; (3) de novo reference-guided viral genome assembly; and (4) automatic report generation to demonstrate NGS data quality, sequence sources, virus composition/abundance, and the assembled genome. This workflow was tested and optimized with datasets featuring SARS-CoV-2 infected patients, as well as pangolin tissues and cells infected by MERS-CoV (downloaded from online databases). The workflow is available on GitHub:https://github.com/haolab410/virus-identification-workflow.

## Implementation

### Data preprocess

The VIW accepts paired-end/single-end next generation sequencing data in one or multiple FASTQ files. Raw data was first checked for quality using FASTQ and FQCHECK. Sequencing quality—i.e., GC-rich content, basic quality of each base, adaptor information, and so on—was returned and organized in the final report. Then, we used Trim-galore to trim the adaptor and low-quality reads with a Phred score of less than 20 and sequence length less than 20 bp. These parameters generally detect more possible viruses with higher coverages.

Clean reads were then aligned to the host genome to remove source sequences (e.g., human sources) using HISAT2. The host genome could be downloaded from the NCBI. We then used SAMtools (specifically the “SAMtools view” command) to filter unmapped reads in order to remove source sequences. For single-end reads, only unmapped (with a FLAG value = 4) strands were retained. For paired-end reads, unmapped strands (with FLAG value = 4), unmapped mate strands (with FLAG value = 8), and unmapped double-strands (with FLAG value = 12) were retained. Unmapped reads were extracted and converted to FASTQ files using bam2fastq before virus identification.

### Virus identification

We employed the FastViromeExplorer [13] to screen the viruses contained in the FASTQ files generated during the preprocess step as FastViromeExplorer was featured by its computational speed and accuracy. Kallisto was used in FastViromeExplorer to align reads to viral reference and estimate virus abundance by screening for exact matches of a short k-mer (31 bp as default) between the reads and the viral reference genomes. The EM algorithm was introduced to assign reads if multiple hits occurred. These ensured an ultrafast speed to detect viruses and accurate estimations of their abundance. In addition, Cr, C0, and Cn were parameters found in the FastViromeExplorer. They were considered to improve virus identification specificity by alleviating artifacts caused by factors such as repeated sequences and low genome coverage. Cr was the ratio of the observed percentage to the expected extent of genome coverage (Cr), which ensure that output viruses would have only one specific repeat region covered. Viruses with a low genome coverage (C0 < 10% by default) or a low total read (Cn < 10 by default) were also discarded.

The trimmed FASTQ files were imported into FastViromeExplorer and short k-mers were mapped with the Kallisto index, i.e., “ncbi-virus-kallisto-index-k31.idx”. Matched virus information was taken from the taxonomy list “ncbi-viruses-list.txt”. These two viral reference files could be directly downloaded from the FastViromeExplorer website. However, since viral genomes increased rapidly every year, we provided the users a shell script (build_index.sh) used to update these viral reference files with the latest NCBI viral data as needed. Our shell script (build_index.sh) could build these viral reference files by downloading NCBI reference viral genomes (ftp://ftp.ncbi.nlm.nih.gov/refseq/release/viral/) and accession2taxid file (ftp://ftp.ncbi.nlm.nih.gov/pub/taxonomy/accession2taxid/nucl_gb.accession2taxid.gz). Further, they could generate the correct format of the requested viral reference files by using the necessary information from downloaded references.

This step output potential viruses, and their abundance detected in the samples. In the next step, the potential viruses were further investigated for genome coverage and mutations.

### Reference-based genome assembly

We defined a three-step reference-based genome assembly for each individual potential virus identified using FastViromeExplorer:

1. Alignment with BWA aln + sampe/samse
  Firstly, we extracted the reads mapped on each potential virus from the output SAM file, generated with FastViromeExploerer, and transformed them into the FASTQ format. Secondly, BWA aln + sample/samse was applied to remap the extracted reads to the individual viral genome. The BWA-mapped reads (SAM format) were extracted, sorted, and de-duplicated for calling single nucleotide variants (SNVs). We did not directly use the alignment output of Kallisto because FastViromeExplorer is a pseudo-alignment and hits depend on a short k-mer (31 bp as default). Although a short k-mer could be conducive to screening out virus’ calculation speed, it is not suitable for genome assembly. Actually, abnormal mutations (i.e., hundreds of SNVs called due to partially aligned reads) popped up when FastViromeExplorer alignments were used.
2. SNV calling using BCFtools
  SNV calling was performed using the multi-pileup file (mpileup) module in BCFtools. De-duplicated reads for each potential virus were indexed using the SAMtools index. Genotype likelihoods were estimated using the mpileup module in BCFtools. SNVs were called via BCFtools.
3. Consensus generation with BCFtools

Consensus was generated using BCFtools consensus, where gaps and positions with low coverage were masked with ‘N’. Variants were output in the form of IUPAC ambiguity codes.

### Report generation

Outputs produced in the previous three steps were finally collected and evaluated to generate a PDF report. The report consisted of four parts:

1. Quality control of the NGS data: consisted of a table summarizing the quality of raw data collected from the outputs of FASTQC and FQCHECK, and figures to visualize the quality results were generated via FASTQC.
2. Identities and compositions of the reads were visualized via a pie-chart using R to clarify the proportions of the low-quality reads, reads from hosts, and reads from viral genomes.
3. Coverage of each viral genome were displayed as follows: (1) a bar-plot showing the counts and percentage of the top five viruses identified; (2) a table summarizing the coverage and species of each virus; and (3) a curve plot depicting the distribution of the counts across the individual viral genome if genome coverage was above 50%.
4. A statistical summary of the assembled viral genomes showed the length and GC content of each virus and consensus genome of each viral genome.

Plots were produced using R and tables were made by collecting summary information generated in previous steps (using shell and Python scripts). Plots and tables were embedded in a brew model, which was then converted from latex to PDF using texi2dvi. We recommend using R 3.5 and higher in the workflow. A full report demo can be found in the supplementary material.

## Results

**Fig. 2:**
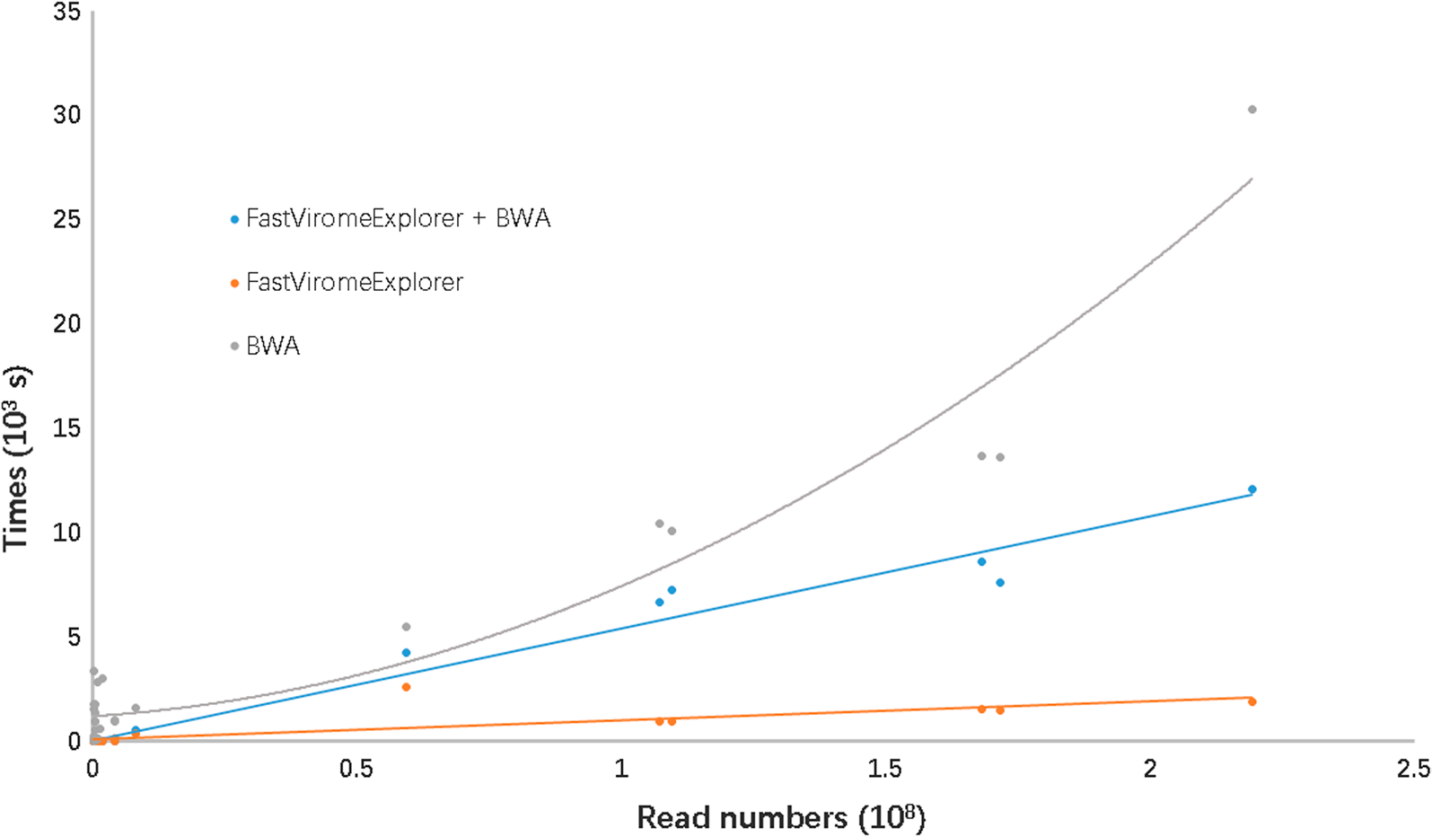
Runtimes needed for the assembling of samples with different sizes by three different methods. The grey line shows the time required for assembly with the bam file contains all the non-host sequences, called it BWA method; the blud line shows the time required for assembly with the sam file contains only the virus sequences detected by FastViromeExplorer, called it FastViromeExplorer + BWA method; and the orange line shows the time required for directly extracted the alignments from the sam output of FastViromeExplorer, called it FastViromeExplorer method. The x-axis is the number of virus reads detected by FastViromeExplorer. And the y-axis is the time used. As the figure shows, when the file size increase, the time used by Bwa method grows polynomially, but the time used by FastViromExplorer + BWA and FastViromeExplorer methods grow linearly

We tested VIW on several datasets downloaded from NGDC (National Genomics Data Center, China) and the EBI-ENA database (Additional file 2). No ethical issue was involved. Since FastViromeExplorer has been proven to be fast and accurate during virus identification, and BWA is a widely used assembly tool, we did not conduct any additional tests to prove the accuracy of the virus identification and assembly. As long as the pipeline reported a virus in the final report, the existence of this virus were separately approved by both software. Our tests focused on the computational speed and accuracy of viral genome assembly in real metagenomics datasets, which have appeared during the COVID-19 viral pandemic. As we described above, our reference guided assembly depended on three steps wherein alignment was the critical one. We attempted three methods to collect the alignments for the individual virus, i.e., FastViromeExplorer, BWA, and FastViromeExplorer + BWA, as well as the estimated quality of the relevant assembled genomes and the computational time to confirm our pipelines. In the first method, which was labeled as FastViromeExplorer, we directly extracted the alignments for each individual virus from the SAM output of FastViromeExplorer using the SAMtools view. Thus, time was saved since no real alignment was performed. It was theoretically fast, but FastViromeExplorer was not a good choice for genome assembly, as is discussed below. In the second method, labeled BWA, mapping between the removed host source reads and the individual viral genome was applied via BWA. FastViromeExplorer + BWA was the method we implemented in VIW, where FastViromeExplorer behaved as a filter in the virus identification step and the alignment was implemented via BWA. We tested the running time of the three methods by running our samples three times with a 24 core CentOS6 machine. We recorded the average times used to assemble all viruses in the samples. Figure 2 shows an overview of the runtimes needed for assembling of samples with different sizes via three different methods. The running time for using the BWA method grew polynomially as the size of the input reads got larger, whereas using our FastViromeExplorer + BWA method, the runtime grew linearly. While the runtime for the FastViromeExplorer + BWA method was faster, the single nucleotide variant (SNV) calling accuracy did not significantly reduce. The running time of the FastViromeExplorer method was even faster than the FastViromeExplorer + BWA method, but the accuracy of the SNV calling went down significantly, as showed in the Tables 1, 2, 3, 4 and 5 below.

### Severe acute respiratory syndrome coronavirus 2 datasets from patients

We first tested our workflow with datasets from COVID-19 patients. In total, 10 samples were collected from three different studies. Sample 1 came from the NGS data of the first SARS-CoV-2 genome, wherein ‘Refseq: NC_045512.2’ was reconstructed (BioProject ID: PRJNA603194, SRR10971381; downloaded from EBI-ENA) [19]. Although only 0.97% of the sequences were identified as viruses, all NC_045512.2 bases were covered with a depth over 200. Neither the FastViromeExplorer + BWA nor BWA methods found a SNV, which was as expected, whereas 1 SNV was found via the FastViromeExplorer. Other viruses were also detected in this sample: Streptococcus phage PH10, with 1.31% coverage; parvovirus NIH-CQV putative 15-kDa protein with 17.57% coverage; and human endogenous retrovirus K113 with 77% coverage. Human endogenous retrovirus K113 existed in the human genome. Streptococcus phage PH10 was a CRISPR (clustered regularly interspaced short palindromic repeats) in human microbiomes [26]. Parvovirus NIH-CQV was thought to be a contamination of the DNA extraction method [27].

Samples 2 (SRR10903401) and 3 (SRR10903402) were two COVID-19 cases from Wuhan (BioProject ID: PRJNA601736; downloaded from EBI-ENA) [20]. As such, 29% and 25% of human sequences were found in these two samples. SARS-CoV-2 was detected in both cases with 99.9% coverage and an average depth above 100 (Table 1). Other viruses were identified, including coliphage phi-X174 and citrus yellow vein clearing virus. Coliphage phi-X174 was found in samples 2, 3, and 7–10 with 100% coverage. It commonly exists in the oral cavity. The citrus yellow vein clearing virus was identified in sample 3 with 86.40% coverage. This virus can cause yellow vein clearing disease of citrus fruits and is spread by aphids from bean-to-bean [28]. No detection of this virus in humans has been previously reported. Therefore, the reason for the virus’ existence needed to be studied. However, a hypothesis was that this virus was caught by the patient when eating citrus fruits, since plant viruses are detected in human samples [29]. We found two SNVs in sample 2 and one SNV in sample 3 using our default (FastViromeExplorer + BWA) assembly method. FastViromeExplorer identified one SNV in sample 2 and no SNV in sample 3. BWA identified two SNVs in sample 2 and one in sample 3 (Table 1). Not surprisingly, the two SNVs found in sample 2 were the same by FastViromeExplorer + BWA and BWA method, but the one SNV found via the FastViromeExplorer method was neither of those two SNVs.

**Table 1:** Overview of virus proportion from Wuhan patients

Sample ID	Host %	Virus %	FastViromeExplorer + BWA (n = 3)				FastViromeExplorer (n = 3)				BWA (n = 3)			
			Time	SD	cov. %	#. SNV	Time	SD	cov. %	#. SNV	Time	SD	cov. %	#. SNV
1	51.18	0.97	1m7s	0m29s	99.90	0	0m47s	0m3s	99.93	1	30m20s	2m41s	100.00	0
2	29.29	58.83	1m42s	0m19s	99.89	2	0m28s	0m4s	100.00	1	1m53s	0m28s	99.92	2
3	25.68	60.98	2m12s	0m6s	99.98	1	0m45s	0m5s	100.00	0	2m35s	0m7s	100.00	0
4	12.22	3.4	0m34s	0m9s	98.94	35	0m15s	0m1s	99.31	1	0m56s	0m5s	98.98	35
5	0.8	0.36	0m10s	0m1s	56.92	25	0m18s	0m2s	66.82	0	4m39s	0m21s	59.37	25
6	43.42	8.82	0m32s	0m10s	87.91	48	0m38s	0m3s	93.74	982	1m20s	0m18s	87,93	48
7	46.94	20.15	2m17s	0m18s	99.98	0	2m7s	0m5s	100.00	2	15m52s	2m36s	100.00	2
8	10.42	9.56	0m47s	0m12s	92.44	41	0m52s	0m4s	99.48	287	17m26s	2m51s	99.36	40
9	19.01	14.65	8m51s	0m54s	99.98	3	6m25s	1m7s	100.00	1	26m55s	3m6s	99.98	2
10	2.02	86.87	71m23s	7m19s	100.00	0	43m33s	1m29s	100.00	0	91m52s	10m44s	100.00	0

Samples 4–10 were downloaded from the NGDC database [21] (NGDC Project ID: PRJCA002202; downloaded from NGDC). The original ID of samples 4–10 were CRR125934, CRR125935, CRR125936, CRR125938, CRR125939, CRR125940, and CRR125941. Sample 5 had coverage under 80% and a depth lower than 2. Sample 7, sample 9, and sample 10 had very few SNVs identified by all three methods. In samples 4, 5, 6, and 8, the FastViromeExplorer + BWA and BWA methods gave similar SNVs numbers (around 20–50 SNVs). This result was also consistent with the one found in the original paper, where 0–51 variants were found [22]. However, the FastViromeExplorer method showed unstable results, either no or very few SNVs, or an extreme large amount of SNVs. Other viruses identified included human endogenous retrovirus K113 with 20% coverage in sample 7 and tobacco mosaic virus with 51.96% coverage in sample 10. Tobacco mosaic virus was a plant virus but had been detected in human samples. Previous studies showed tobacco mosaic virus could stay in mice cells for more than 15 days [29]. Since the COVID-19 outbreak lasted only several months, an extremely large number of SNVs suggested a low accuracy of the method.

As shown in sample 1–10, the FastViromeExplorer method was fastest when running on the same sample because it did not require realignment with BWA. However, the low accuracy of this method suggested that assembly with the output of the FastViromeExplorer was not appropriate. The FastViromeExplorer + BWA method took a little more time than the FastViromeExplorer method, but was much faster than the BWA assembly method, and gave a more reliable assembly result.

### Transcriptomic analysis of the Novel Middle East Respiratory Syndrome Coronavirus (Human, MRC5 cells)

A test on five samples from human MRC5 cell culture with MERS-CoV infection was also implemented to further inspect the workflow performance (BioProject ID: PRJNA233943; downloaded from EBI-ENA). Samples 11–15 represented SRR1192017, SRR1191695, SRR1191876, SRR1191783, and SRR1192321. Samples were prepared by infecting human lung-derived MRC5 cells with MERS-CoV for 48 h, and profiled by high throughput sequencing (NCBI GSE56192 summary). In these cases, human sequences accounted for around 32.68–40.14% of total reads, and viral genome proportions were similar to the one in SARS-CoV-2 patients’ samples. Two MERS-CoV sequences were found in all five cases with coverage over 98% and average reads depth above 10,000, as shown in Tables 2 and 3. Coliphage phiX174 with 100% coverage was detected in all samples. Abelson murine leukemia virus with coverage less than 1.7% was detected in samples 11, 12, 14, and 15. It should be a lab contamination since these samples were infected in the lab. Human endogenous retrovirus K113 with coverage of about 80% in all samples were also found in all samples.

**Table 2:** Overview of virus MERS NC_019843.3 in MERS infected MRC5 cells

Sample ID	Host %	Virus %	FastViromeExplorer + BWA (n = 3)				FastViromeExplorer (n = 3)				BWA (n = 3)			
			Time	SD	cov. %	#. SNV	Time	SD	cov. %	#. SNV	Time	SD	cov. %	#. SNV
11	32.68	65.73	111m12s	3m15s	100.00	5	16m15s	2m7s	100.00	4	166m38s	9m0s	100.00	6
12	40.07	58.10	121m17s	17m3s	99.90	5	16m16s	2m50s	100.00	5	163m10s	17m3s	100.00	5
13	38.45	59.85	144m15s	12m53s	100.00	5	25m36s	3m38s	100.00	4	228m17s	17m14s	100.00	5
14	40.14	57.96	123m19s	16m10s	98.86	5	25m12s	4m13s	100.00	4	234m6s	13m53s	100.00	5
15	32.23	66.20	193m33s	7m25s	100.00	4	31m40s	4m1s	100.00	4	540m25s	54m25s	100.00	5

**Table 3:** Overview of virus MERS NC_038294.1 in MERS-CoV infected MRC5 cells

Sample ID	Host %	Virus %	FastViromeExplorer + BWA (n = 3)				FastViromeExplorer (n = 3)				BWA (n = 3)			
			Time	SD	cov. %	#. SNV	Time	SD	cov. %	#. SNV	Time	SD	cov. %	#. SNV
11	32.68	65.73	111m12s	3m15s	99.99	106	16m15s	2m7s	100	31	166m38s	9m0s	100	99
12	40.07	58.1	121m17s	17m3s	100	106	16m16s	2m50s	100	21	163m10s	17m3s	100	96
13	38.45	59.85	144m15s	12m53s	100	105	25m36s	3m38s	100	24	228m17s	17m14s	100	99
14	40.14	57.96	123m19s	16m10s	98.83	99	25m12s	4m13s	100	19	234m6s	13m53s	100	96
15	32.23	66.2	193m33s	7m25s	100	105	31m40s	4m1s	100	31	540m25s	54m25s	100	99

The numbers of SNVs in these five samples were similar, since human cells were infected by the same laboratory MERS-CoV strain in vitro. As shown in Table 3, MERS-CoV strain NC_019843.3 had about five SNVs when using all three assembly methods. MERS-CoV strain NC_038294.1 had about 105 SNVs when using the FastViromeExplorer + BWA assembly method, about 99 SNVs when only using the BWA assembly only, but less than 31 SNVs when only using the FastViromeExplorer assembly. The FastViromeExplorer assembly method showed a larger standard deviation in the number of SNVs among the five samples compared with the STD from the other two methods.

Since these samples were over 8 Gb, it took much longer to run. The time used by the FastViromeExplorer assembly method was much shorter than the FastViromeExplorer + BWA method, and was much shorter than the BWA assembly method in all five cases.

### Virome of dead pangolin individuals metagenome

To test whether our workflow could be applied to different host species, we ran our workflow on pangolin samples because reports showed that SARS-CoV-2 might derive from pangolin [25]. Thus, we expected to find this virus in the pangolin samples. Metagenomics data from 11 pangolin lung samples were downloaded from the NCBI database (BioProject ID: PRJNA573298; downloaded from EBI-ENA). In Liu’s paper [23], 40–50% host sequences were found using BLAST-based aligning (BWA). We found 20% via HISAT2 in our workflow. A fitting explanation was that there existed unknown alternative splicing isoforms in the pangolin genome since pangolins were not as thoroughly studied as model organisms. Viral sequence proportions found in samples were low in both our workflows (0.31–4.51%) and Liu’s paper (lower than 0.5%). SARS-CoV-2 was found in two lung samples (lung07 and lung08) via our workflow, but with low viral genomes coverages, as shown in Table 5. In Liu’s article, a coronavirus was only found in these samples [23]. We also found 7 lung samples with the Sendai virus, as shown in Table 4, though only one read from Sendai virus was found in sample lung11. Six samples with Sendai virus were reported in Liu’s study. There was no coverage data shown in Liu’s study, but coverages of viruses in this dataset were low compared to the previous two datasets. Using the FastViromeExplorer + BWA method, Sendai virus sequences were found in 9 lung samples, but only 7 samples assembled this viral genome successfully via BWA with at least one read. The reason for this might be that FastViromeExplorer (Kallisto) was able to detect part of the read (31 bp), but the remainder of the read might have failed to match the virus of interest, indicating that there was insufficient evidence to prove the existence of the virus. Therefore, the viruses that were detected using FastViromeExplorer with 0% output coverage of their viral genomes when running BBmaps (no read assembled for the virus) might be a false positive result and would not be presented in the final report. The information of these viruses was saved in an auto-created file named “fve/FastViromeExplorer-final-sorted-abundance.tsv”. The Sendai virus was not found in sample lung13. The times showed in the table refers to the total assembly time of all viruses in the sample. Another virus (Parus major densovirus) was detected in lung13.

**Table 4:** Overview of Sendai virus in pangolins’ lung samples

Sample ID	Host %	Virus %	FastViromeExplorer + BWA (n = 3)				FastViromeExplorer (n = 3)				BWA (n = 3)			
			Time	SD	cov. %	#. SNV	Time	SD	cov. %	#. SNV	Time	SD	cov. %	#. SNV
Lung01	8.09	1.49	0m21s	0m16s	0	0	0m6s	0m0.2 s	53.66	222	16m8s	3m28s	1.94	0
Lung02	19.25	3.67	0m21s	0m5s	6.37	0	0m21s	0m1s	72.2	861	43m57s	10m30s	10.47	4
Lung03	8.32	2.16	0m34s	0m17s	0	0	0m7s	0m1s	22.19	23	16m25s	1m14s	0	0
Lung04	12.29	2.23	0m18s	0m20s	8.66	6	0m5s	0m0.1 s	75.27	915	9m31s	1m2s	9.91	6
Lung07	9.09	0.63	1m21s	0m30s	5.67	0	0m22s	0m3s	61.76	469	56m48s	7m15s	5.88	9
Lung08	11.07	0.67	0m43s	0m26s	5.16	0	0m19s	0m2s	72.07	743	25m46s	3m38s	7.36	0
Lung09	8.03	0.31	0m22s	0m4s	9.06	0	0m5s	0m0.2 s	74.32	957	30m9s	2m41s	9.33	0
Lung11	9.32	0.69	0m47s	0m39s	0.97	0	0m5s	0m1s	18.65	31	23m5s	3m11s	0.97	0
Lung12	6.82	1.03	0m1s	0m0.1 s	NA	NA	0m2s	0m1s	NA	NA	0m0.1 s	0m0.05 s	NA	NA
Lung13	21.76	4.47	0m21s	0m19s	NA	NA	0m13s	0m1s	NA	NA	10m22s	1m39s	NA	NA
Lung19	7.47	3.82	0m23s	0m2s	13.23	7	0m14s	0m1s	87.86	1013	47m59s	4m1s	14.5	7

**Table 5:** Overview of SARS-CoV-2 virus in pangolins’ lung samples

Sample ID	Host %	Virus %	FastViromeExplorer + BWA (n = 3)			FastViromeExplorer (n = 3)			BWA (n = 3)		
			Time	cov. %	#. SNV	Time	cov. %	#. SNV	Time	cov. %	#. SNV
Lung07	1.32	0.62	1m21s	7	43	0m22s	24.88	469	56m48s	8.86	41
Lung08	2.05	0.65	0m43s	11.53	37	0m19s	51.58	743	25m46s	13.87	37

As shown in Table 4, by using the FastViromeExplorer + BWA alignment method and the BWA only alignment method, very few SNVs were found in the Sendai virus. The FastViromeExplorer method showed an abnormally large amount of SNVs. The total length of the Sendai virus genome was 15,384, but 1013 SNVs were found in sample lung19. The same trend was also found for SARS-CoV-2 virus (Table 5). The FastViromeExplorer + BWA method and the BWA method output a reasonable amount of SNVs (around 40), whereas the FastViromeExplorer method detected hundreds of SNVs. With a relatively small amount of viral genome sequences in the samples since the coverage of the virus was very low, the FastViromeExplorer assembly method still ran fastest, followed by the FastViromeExplorer + BWA method, whereas the BWA assembly method took a much longer time to run. Lung12 took only 1 s to run because no potential virus was found in this sample.

### Trimming effect

We also ran our workflow on datasets with different trimming conditions to test whether the trimming strategy would affect virus detection. We tested our datasets with q = 20, length = 20; q = 30, length = 30; and q = 40, length = 50. The meaning of q = 20 was that, for any base, if its Phred score was less than 20 (meaning probably 1 incorrect base call in 100 bp reads), it would be trimmed off, and the meaning of length = 20 was that, after the base trimming, the whole read would be trimmed off if the total length of a read was less than 20 bp. The results showed that most viruses can be detected with q = 20 and length = 20. The disappearance of viruses in the other two conditions could be due to the cutoff of those virus sequences in the samples. Especially when we used q = 40 and length = 50 to trim the sample reads, all sequences in samples 1–10 were trimmed off, as shown in Additional file 3. The coverages of our samples went down or remained unchanged in most cases when increasing the Phred score and read length. In some cases, virus coverages could increase by about 1% in the condition of q = 30 and length = 30 compared with the condition of q = 20 and length = 20. However, some viruses were not detected when the condition increased to q = 30 and length = 30, as shown in sample 15. Although these viruses had coverages lower than 20%, implying that these might be due to mismatching, we still hoped our pipeline could detect all possible viruses in the samples to provide more information to users. On the other hand, trimming is necessary to remove adaptors and low-quality reads to avoid false positive SNVs. Therefore, we used q = 20 and length = 20 as the trimming parameters in our pipeline. By choosing these parameters, our pipeline could access all kinds of reads with different qualities since the sequence qualities were usually above the provided screening criteria.

## Discussion

We tested our workflow, VIW, with samples of SARS-CoV-2 from human and pangolin to show its capability of handling sequencing data for multi-species. We test the workflow with samples of SARS-CoV-2 and MERS-CoV from human cell samples to show its capability to identify different viruses in samples. This workflow can be used widely in clinical studies. For example, it can be used for the SARS-CoV-2 epidemic as additional evidence for diagnosis. The workflow is very flexible and can be used on various NGS datasets for various purposes. Although there are other applications for virus detection purposes—e.g., toolset FastV, released by Zhang’s team [24]—our workflow is the only workflow that outputs information about virus proportion and generates viral genome sequences for each identified virus in each sample. The workflow design also enables us to identify a bacteria genomes step for future optimization. A web tool is in creation for easier usage now.

Because we used reliable software to filter host sequences, removed adapters and low quality reads in the preprocessing steps, and identified viruses during the virus identification step, our biggest concern for the presented workflow is the accuracy of the virus assembly step. As for virus sequence alignment, although FastViromeExplorer did a good job identifying viruses, it cannot be used as a direct alignment method, since it might bring unstable assembly results, i.e., extremely large or small number of SNVs. BWA is a reliable software for alignment, because it considers a full length alignment of the reads to the reference genome. However, it takes a long time when the input is large. We therefore combined FastViromeExplorer (Kallisto) and BWA to fulfill the two tasks, i.e., virus detection and genome assembly, wherein it worked as a virus detector and a filter in order to reduce the amount of sequences need to be aligned by the BWA. On one hand, our workflow retained its alignment accuracy by using BWA. On the other hand, our tests demonstrated that the running speed of our default method was faster than using the BWA assembly directly. Therefore, even though we provide other alignment methods on GitHub, we highly recommend our default method, as it is suitable for most NGS data. In addition, the human-friendly report generated in our workflow systematically presents results in each step, and makes it more convenient for researchers to understand their NGS data. In summary, the advantages of our workflow are that it generates results quickly and accurately, and the results are easy to understand.

Although our workflow is sensitive enough to detect viruses with only one read present in the sample, which happened in the Sendai virus for sample lung11, our workflow lacked a method to decide whether a virus actually existed in the sample. The workflow kept the Sendai virus in its report, as identified virus in sample lung11. Therefore, our workflow detected 7 pangolin lung samples with the Sendai virus. However, the fundamental study suggests that only 6 samples contained this virus. Since we only used online datasets for our tests, we could not verify this. Thence, further tests should be done to clarify the minimum coverage needed to determine the existence of a virus. Another problem of this workflow is that since it assembled viral sequences using known viral genome for reference, it could not assemble unknown viruses. For instance, pangolin samples lung7 and lung8 both reported the contamination of SARS-CoV-2, but with only 7.00% and 11.53% total coverage. It is not clear whether this virus found in the human being was SARS-CoV-2. If it is an unknown coronavirus, our workflow can only define it with its closest relative, but not a novel virus. In another words, when the coverage of a virus produced in our workflow is low, further tests and experiments are requested to identify the virus. This could be done with further experimental verification, or by testing more samples with our workflow.

## Conclusion

In conclusion, our workflow, VIW, can be used to identify viruses contained in a host genome. By running this workflow, users can obtain the following: a table that lists all potential viruses in their samples; a file with SNV information for each identified virus; a FA file with assembled viral genome; and a report visualizing the quality and composition of raw data, and information on the coverage and taxonomy of identified viruses. The workflow can be used as the first step to study viruses, where virus sequences are extracted and assembled from NGS raw data. In addition, the workflow can be used for clinical diagnoses.

## Supplementary Information

**Additional file 1**: An example of the final report generated by our pipeline.

**Additional file 2**: Sample Sources.

**Additional file 3**: Viruses identification among different reads quality cut off.

## Data Availability

All datasets were downloaded from online databases, and were detailed in Additional file 2.

## Abbreviations

NGS: Next-generation sequencing
MERS: Middle east respiratory syndrome
R0: The basic reproductive number
Gene-based: Amino acid profile gene-based approaches
Reference-based: nucleotide profile reference-based approaches
Cr: Expected extent of genome coverage
C0: Low genome coverage
Cn: Low total read (single nucleotide variants (SNVs)
mpileup: Multi-pileup file module
NGDC: National Genomics Data Center, China
CRISPR: Clustered regularly interspaced short palindromic repeats
BWA: BLAST-based aligning

## Availability and requirements

Project name: Virus Identification Workflow. Project home page: https://github.com/haolab410/virus-identification-workflow. Operating system(s): Linux. Programming language: Shell, R, Python. Archived version: 10.5281/zenodo.3968324. Other requirements: kallisto 0.43.1 to 0.45, samtools 1.6 or higher, R 3.5 or higher. License: Open Access This article is distributed under the terms of the Creative Commons Attribution 4.0 International License (https://creativecommons.org/licenses/by/4.0), which permits use, duplication, adaptation, distribution, and reproduction in any medium or format, as long as you give appropriate credit to the original author(s) and the source, provide a link to the Creative Commons license, and indicate if changes were made.
